# Statistical Reports of the Health of the Navy, for the Years 1830, 1831, 1832, 1833, 1834, 1835, and 1836, in the South American, West Indian, North American, Mediterranean and Peninsula Commands

**Published:** 1840

**Authors:** 


### Medico-Chirurgical Review,

### No. LXVI.

[No. 26 of a Decennial Series.]

JULY 1, to OCTOBER 1, 1840.

### Statistical Reports of the Health of the Navy, for the Years 1830, 1831, 1832, 1833, 1834, 1835, and 1836, in the South American, West Indian, North American, Mediterranean and Peninsula Commands. Ordered by the House of Commons to be Printed. 1840.

The Returns from the Army, published by Major Tulloch, are now followed by similar Returns from the Navy. We were confident that the able physician at the head of the Medical Department of the latter Service would take care to render the Reports transmitted to the Admiralty as available as possible to the interests of medicine and the public, and we have not been disappointed. There is no man who would do more in the exercise of his official authority for the promotion of science than Sir William Burnett.

The returns before us are drawn up by Dr. Wilson, who informs us of the circumstances under which it was commenced and has been carried on. It appears the inquiry was begun towards the close of the year 1836, by order of the Lords Commissioners of the Admiralty, and had been in progress some months, when, on the sudden death of the officer first appointed to the duty, Dr. Wilson was called on unexpectedly to perform it. When he had fairly entered on it, and considered the ends in view, with the best method of obtaining them, it appeared to him that the plan adopted by his predecessor was in some points defective and ill-devised, and that to pursue it, without addition, or correction, would, in a great measure, defeat the object of the undertaking; because it must lead to unsatisfactory, or worse, to erroneous conclusions. It was defective, inasmuch as it was not proposed to trace disease from ships rigidly to, and through hospitals, thereby omitting much of the mortality and invaliding; and the method of computing the mean force of squadrons was erroneous, raising it, and in some instances by a large amount, above the number employed. The effect of omission in the one case, and addition in the other, would have been to give an exaggerated view of the health of the navy, and to represent it as being more exalted than it appears to be by the following Reports, and than it really is. In these circumstances, it was necessary to begin the work anew, and all the previous labour, however unwillingly the sacrifice was made, was lost. This statement is given, not with a view of disparaging another, who is gone, but to account, in some measure, for the time which has elapsed between the ordering, and presenting the Reports.

This much seems due to Dr. Wilson. He explains, in an INTRODUCTION, the mode in which the Reports are collected, and, consequently, the degree of credit that attaches to them.

It appears that, medical officers, whether surgeons, or assistant-surgeons, while in charge of the health of ships' companies, are required, by Admiralty instructions, to transmit to the Physician-general of the Navy the following documents, relating directly to such diseases and injuries, and their results, as may occur in the ships to which they respectively belong, viz. a Daily Sick Book, Monthly or Three Monthly Nosological Returns, and a Journal of Medical and Surgical Practice.

The first exhibits, in separate columns, the date of entry on the surgeon's list, the patient's name, age, quality, nature of disease or hurt, date of discharge from the list, and the issue of complaint whether in cure, removal to hospital, invaliding, or death; it is transmitted annually.

The second gives a comprehensive view of the first. In it names of patients are omitted, but all cases of disease, and injury, are contained and arranged in a tabular nosological form. This return is transmitted at the end of every month from ships on the home stations, and at the end of every three months from ships on foreign stations, except in cases of prevalent, or of unusually fatal disease, when more frequent communication is ordered. It exhibits in classes and orders, the number of cases added to the list, the number discharged to duty, the number sent to hospital, the number dead, the number invalided, the number on the list at the time it is sent, the number confined to bed, the number convalescent, the number of objects for survey, and the number remaining from the previous return. The Cullenian method of arrangement is that adopted in the navy. In each Nosological Return it is required that remarks be appended to the figures on health and sickness, and whenever it can be done, on the appreciable causes.

The third return contains a detailed account of the symptoms of disease, and injury, and of the treatment instituted for their cure. It is, in fact, a journal of the medical transactions of the ship, showing the nature, progress, and management of all important complaints, till the subjects of them shall be cured, removed to hospital, invalided, or die. Attached to it is a tabular abstract of all the cases therein treated, with their terminations; it is concluded by general remarks on the matter it contains, and is transmitted annually. And it is especially desired, when very prevalent or fatal forms of disease happen in ships, that they should be traced to their sources, and accounted for, if possible. It is also directed that states of weather, degrees of temperature, the general interior economy of the ships, and whatever else may appear to conduce to health, or to induce disease, be specified.

The tables have been deduced from these documents, together with returns from hospitals, and sick quarters.

At present there are only five naval hospitals, viz. at Portsmouth, Plymouth, Malta, Jamaica, and Bermuda, and two marine hospitals, those of Chatham and Woolwich, for the reception of seamen; but sick seamen and marines are received into all military and colonial hospitals, in such places as do not contain naval hospitals. Besides those institutions, sick quarters are established at fifty stations for the accommodation of sick and hurt seamen and marines, where surgeons are appointed to attend such as may require the quietness and comforts of shore, procuring them suitable lodgings, diet, medicines, &c; hence the appellation "sick quarters." All these fifty sick quarters are in the United Kingdom, and have reference to the tables of the Home Stations only. During part of the time embraced by this Report there was an additional naval hospital at the Cape of Good Hope.

In peace, no physicians of the fleet, or other superintending medical officers are employed afloat; and consequently no synthetical, comprehensive accounts of disease, and the results of disease, can be sent to the physician-general from the various naval commands. The consequence is that the reports are multitudinous, minute, not unfrequently broken, and collated and summed up with no little labour, and indeed difficulty. Absolute accuracy, under such circumstances, can hardly be expected, but the deviations from it have not been material. But although there can be no material incorrectness respecting the ratio of mortality, errors are admitted by Dr. Wilson to be likely in the case of invaliding. He believes he may have rated it too high. Dr. Wilson enters at some length into the circumstances that interfere with the results of calculations. These are neither few in number, nor inconsiderable, but we must refer those who would wish to be acquainted with them to the Report.

A table is furnished containing the *allowance of provisions* in the fleet. It is rather long, and we think that some of the general observations which follow will sufficiently indicate the more important points.

*Diminution of the Supply of Spirits.*—Previously to 1825 half-a-pint of spirits, when spirits were issued, was allowed to every person serving in the fleet; at which time a salutary and judicious change was introduced, by the reduction of the spirits to a quarter of a pint daily, and the allowance of tea, or coffee instead. The practice formerly was, to divide the half-pint of spirits into two equal parts, one of which was issued at dinner-time, the other in the afternoon; now, instead of the afternoon allowance of spirits, tea or coffee is issued, and proves a safe, healthy, and satisfactory article of diet. When the change was introduced, it was apprehended by some that the seamen, if they did not resist, would be greatly dissatisfied with it, their love of grog being considered paramount to all considerations. It was, however, introduced without disturbance, or general complaint; in a short time it became liked; and, now, it is believed, that the majority of the men serving, if it were put to them, would prefer the present to the former system. It is certain that the change has acted, and will act yet more beneficially; for it is unnecessary to state, that one of the most active causes of disease and insubordination, with all its mischievous results, has been the intemperate use of spirituous liquors. Well may Dr. Wilson add, that we cannot doubt that increased temperance, aided by other means of improvement, will eventually give the navy a force, organic, moral and intellectual, much greater than any it has hitherto possessed.

The naval rations are abundant in quantity and excellent in quality. It cannot be questioned that these circumstances contribute largely to the high degree of health now enjoyed in the Royal Navy. Formerly a ship of war was, on many accounts, an object of aversion; destructive disease, under various forms, being one. Scurvy, putrid ulcer, malignant dysentery, and fever, allied to that of gaols, suddenly swept off the greater portions of many ships' crews, and well nigh depopulated fleets. Many causes, no doubt, concurred to occasion those maladies; but in the production of the first and worst, the most fertile and constant, was insufficient nutrition, resulting from scanty, and unwholesome food. It is matter of surprise, not less than of regret now, that the pervading agency which led to such disastrous results on health in those days, should not have been known, or, if suspected, was not counteracted. Till the year 1796, scurvy continued to infest the fleet, to cripple its power by the number it affected, and, in many instances, to produce a large amount of mortality.

In 1797 the victualling was changed, greatly improved, and strictly regulated, and consequent immediately to the change, the health of seamen improved strikingly. Scurvy, typhoid fever, dysentery, and ulcer, which, up to the period of change, had produced great havoc, became comparatively rare in occurrence, and light in impression. Some ships, in particular circumstances, suffered from one or more of those diseases; but the sweeping epidemics of former years, which often rendered individual ships, and sometimes entire fleets, totally ineffective, became unknown. Since 1797 various improvements have been introduced into the victualling of the navy, such as giving cocoa in lieu of gruel (burgoo) for breakfast, issuing salt meat at a much earlier period, after being cured, the supply of better articles, and the substitution of tea for the afternoon allowance of spirits; and with every improvement in those respects, as a general result, has there been further improvement in health, till the *four forms* of disease, at no distant date so destructive, are scarcely known, except by name. Endemic fever, the effect of extraneous agency, in particular localities, sometimes prevails extensively and fatally; acute inflammatory dysentery is not uncommon, nor frequently without serious results; and ulcerative disease is any thing but rare, and is often extremely troublesome.

Previously to the year 1797, the nutriment supplied by public rations to seamen and marines, was at least a third less than it is now. What effect this must have had may be guessed with ease.

"Palatable water, in sufficient quantity, is essential to comfort, and influential on health; and in no article, at least in the manner of keeping, and preserving it, has there been greater improvement than in this indispensable one, in recent times, in ships of war. When water was kept in casks, it became slightly fœtid, from the disengagement of hydrogen, in a few days, and, in a fortnight or three weeks, so loathsome, as to be swallowed with repugnance, even when called for by urgent thirst. The progress of decomposition, and its nauseating results, were especially rapid and offensive, when the water was most pure, at least, when it contained the smallest portion of mineral admixture, and the temperature was high. When the solid food at sea consisted almost exclusively of very salt beef and pork, biscuits long baked, and puddings made of salt suet and flour, the desire for, even the necessity of abundance of water, was great. No one who has not felt it can imagine the distress that was often endured, within the tropics, setting aside the effects on health, from the intense thirst thus excited, and the only means available for quenching it. Water so putrid, and offensive, often so thick, and green from vegetable admixture, and decomposition, and emitting so strongly the fætor of rotten eggs, as to disgust at once the sense of smell, and of taste.

Happily all those evils and inconveniences are banished from the Navy, by the substitution of iron tanks for water-casks. Water suffers no change in these iron vessels, however long kept, at least no change in itself, from decomposition. The metal becomes oxydised to a certain extent, and the oxyde in the interior of the tank mixes with the water, but, from its weight and insolubility, falls to the bottom, and does not, except in stormy weather, discolour the water, till the tank is nearly empty. When the water is taken from the tank in stormy weather, or from the bottom, it has a brownish colour, on account of a portion of the oxyde of iron being suspended in it, the greater part of which soon falls to the bottom of the vessel into which it has been drawn. It is not tainted with any thing offensive either to the palate, or the nose. There is no reason to suppose that the slight chalybeate admixture is injurious to health; it may be in such minute portions beneficial." xiv.

Dr. Wilson properly recommends great care in procuring water originally good. To save a little expense bad water has been taken—an injudicious as well as cruel parsimony.

The diet of the sick seamen in the naval hospitals is unexceptionable. Dr. Wilson gives a table which exhibits it; and he adds—In ships the diet of the sick is as nearly the same as the circumstances in which they happen to be placed will permit. When long at sea, fresh meat in any quantity, except poultry, cannot be procured; but preserved meats, and soups, good substitutes for fresh beef, and vegetables, are supplied, and are made available, with many other comforts, for the support of the sick and convalescent.

It is due to the present Board of Admiralty, and the Physician-general, by whom it was proposed and recommended, to state, that the diet of the sick at sea has been much improved within the last five years. Previously, though preserved meats, tea, sugar, sago, rice and barley, were supplied, there was no money fund by which soft bread, potherbs, poultry, spices, milk, porter, &c. could be procured; and consequently in many cases of sickness, and more of convalescence, many things were wanting, and the privation was severely felt by the medical officers, as well as the patients, of essential importance to the speedy restoration of health, if not to the absolute preservation of life.

From diet, the Reporter passes to the *general economy* of ships of war, under the heads of modes of employment, of berthing, of cleaning, and of ventilating. It is both interesting and necessary to become acquainted in some degree with life on board ship, in order to appreciate the causes of the health or of the diseases of sailors.

1. *Employment.*—Seamen, we quote the Reporter, and embarked marines, excepting those employed as sentries, are divided into two watches, on which alternately the working of the ship devolves. Such is the practice now, though at one time it was not uncommon to divide the working force of ships of war into three watches. Each method has certain advantages, and drawbacks. Three watches give the men longer intervals of rest, which, during the night at least, is desirable, but necessarily reduce the strength of the party in charge to an extent, which might be, especially with reduced complements, dangerous, and must be fatiguing. When, as is common, if not universal now, the men are divided into two watches, they perform alternately, during four hours each, the sailing operations of the ships. Each party has four hours' duty, and four hours' rest, or, as is said, is four hours on deck, and four hours below, excepting the four hours between four and eight o'clock at night, which are divided into two half, or, as they are called by sailors, dog-watches. The effect of this break is to alter constantly the periods of labour and rest to each party; so that the men who have the first watch one night, have the second watch the next night, and so on till the circuit be completed.

In this way, though there is sufficient time for rest, it is never long continued, and sleep is broken into short periods. Whatever effect such a division of labour and rest may have on constitutional vigour, though it is perhaps the best that can be devised, it probably contributes much to the frequency of some affections, such as catarrh, and rheumatism, to which seamen are so subject. At midnight, or four o'clock in the morning, the men relieving the deck rush from their beds into the open air, often very inadequately covered, perhaps perspiring profusely, and pass in an instant from a highly heated, and debilitating, to, it may be, a really cold, always to a comparatively cold atmosphere. In such circumstances it is to be expected that catarrhal, rheumatic, and other affections, traced to sudden, reductive changes of temperature, should be numerous. These remarks apply chiefly to ships at sea. In port the number of men kept on deck lessens with the increasing safety of the anchorage, till, in ships moored in secure harbours, all hands, excepting the officer, petty officers, and sentinels in charge, may pass the whole night in bed.

2. *Berthing.*—Seamen and marines are berthed, that is, mess and sleep, on what is called the lower deck of all ships under first rates, in which they occupy the middle, as well as the lower deck. By lower deck, in frigates, and all smaller vessels, is meant the lowest, or that covering the holds, and store-rooms; but in ships of the line it means the lower gun-deck, and has the orlop deck between it and the holds. There is, therefore, material difference between the parts of the vessel in which the people live in ships of the line, and in frigates, and smaller craft. In the former there are ports which can be kept open in moderate weather; in the latter there are only small apertures—scuttles—close to the water, which cannot be kept open except in an absolute calm at sea, and very fine weather even in harbour. Hence, as well as from the intervention of a deck between the crew and the holds, ships of the line have more means of ventilation, and are generally better ventilated than frigates, and smaller vessels.

In all ships, from the number embarked, and the space available for what may be called domestic purposes, viz. for eating, and sleeping, the men are, from necessity, much crowded between decks, the least relatively in three deckers, for in them the crews have two decks to live on, and the numbers are not augmented in the ratio of the space. Yet it is not clear that the benefit thus obtained, by the reduction of pressure, is not counterbalanced by the increased volume of heated and vitiated air in which the men live. So far as it can be accomplished, even at the expense of increased crowding, it appears to be better to berth crews, at night, in one tier, than in two tiers; for at sea, and even in harbour, except in very moderate weather, the ports must then be closed, and fresh air can be conveyed only by the hatches, to the sleeping places; when they are double, adequate ventilation, supply of pure and evolution of impure air, with resulting reduction of temperature, is much more difficult than when they are single. The difficulty does not depend on the increased volume of heated and deteriorated air thus produced, but also and principally on its being intersected by a deck; for the pure air conducted down the hatches can be directed to one division only, leaving the other in a state of increasing impurity, the means of admitting air to it being extremely imperfect, during four, six, or seven hours at a time.

The usual space between the suspending points (clues) of the hammocks, is from 14 to 18 inches; so that when they are extended by the beds, their bodies are in contact. The effect is to bring the bodies of the men into contact, in greater or less number, according to the size of the ships. When at sea, with a watch on deck, the accumulation and pressure are reduced by a half, but when in secure harbours, 500 men, perhaps, sleep on one deck, their bodies touching each other, over the whole space laterally, and with very little spare room lengthways.

The *comfort* of this within the tropics is conceivable. Yet the prejudicial effect on health has proved scarcely appreciable, and, as Dr. Wilson observes, the actual salubrity of ships shews that air may be contaminated to a certain extent, for a considerable period, without producing any decidedly deleterious effect, immediate or remote, on the persons breathing it. The fact we presume to be, that the foul air breathed for a short time is more than compensated by the fresh air inhaled for the greater portion of the twenty-four hours. How different with regard to *air*, after all, is the sailor, crowded though his berth may be, and the pale inhabitant of a London alley.

3. *Cleaning.*—This is carried ad extremum in the navy. The ordinary methods employed are washing, wet, and dry stoning. In the first, large quantities of sea-water, with friction by brushes, is used; in the second, a small quantity of water is poured on the decks, which are then diligently rubbed with smooth, flat stones, generally of sand-stone, designated holy-stones by the seamen, for the purpose of removing stain-spots, grease, &c. In the third, the same kind of stones are used for rubbing, but, instead of water, they are applied directly over a small portion of sand, cold or heated, which has been scattered on the decks. There is a fourth method sometimes employed, called sprinkling and scrubbing, in which the decks are slightly wetted, and rubbed with brushes, or dried by cloths, so far as they can be dried by such means. The selection from, or alternation, in various proportions of these methods, is left to the determination of officers in command.

" In each of them," Dr. Wilson continues, " except that of dry stoning, more or less water is employed, a portion of which, whatever pains be taken to remove it, is absorbed by the planks, or lodges in their crevices, to be dissipated in the course of considerable time by evaporation. In ships where daily washing, or wet cleaning is practised, the decks are never thoroughly dry; the evaporating process which carries off the retained moisture of one morning, is not completed till the washing operations of another morning are begun. Frequent washing of the upper, and upper gun deck, in ships of the line, is perhaps unobjectionable, but as applied to the lower and orlop deck, and the decks of other vessels where the people eat and sleep, its use should be restricted to the demands of necessity. Evaporation, especially in low decks, and low degrees of temperature, goes on slowly, and therefore long; in hot climates it is of course more rapid, and sooner completed; but in either case there is strong reason to conclude, as well from observation, as the nature of the things, that the effects on health are injurious, sometimes highly so."

Catarrhal and rheumatic affections would, of course, be likely to occur, and Dr. Wilson thinks there cannot be much question of their power of exciting many of the inflammatory affections of the lower extremities which give rise, in some ships, to much mischief.

Cleaning decks by dry stoning is free from these objections, and is therefore, in these important respects, preferable. Its power to remove impurities, especially those which discolour the planks, and are so far offensive to the eye, is not so complete. Besides, when very friable (sometimes calcareous) stones are employed, a good deal of dust is disengaged in the process, which irritates the eyes, settles on the clothes, and insinuates itself into the chests, bags, &c., and is therefore to a certain extent annoying. But, as the Reporter observes, these are small evils, and should not be weighed against the more serious ones of frequent washing. Spotless whiteness of the decks is not necessary, and may be bought too dear.

The holds, wells, and spaces under the limber boards, in which accumulations of extraneous decomposable matters are more likely to happen than on the open decks, should be closely looked to; and they generally are. Personal cleanliness is strictly enforced, and, indeed, for criticism or for alteration in any respect. But high as is the present standard of health, Dr. Wilson anticipates its being raised even higher, and would stimulate all concerned to exert themselves to the uttermost.

*Employment.*—We need not dilate on the beneficial influence on health of a cheerful condition of mind. Until lately this was sought in physical rather than in intellectual enjoyment; and the dance, and song, and athletic sport were all that was thought necessary for Jack. But the intelligence of the age has penetrated the strong sides of the line-of-battle ship, and the sailor must be looked upon, and is, as a moral engine and a moral agent, as well as a mere machine. We most cordially concur in the following remarks of the Reporter.

"Little was attempted till within the last 20 years, except the appointment of chaplains, and schoolmasters to certain ships; and, setting aside the performance of divine service on Sunday by the former, the value of which is not questioned, it is no breach of truth or charity, to say that little was done by either for the working sailor, or marine. Little pains were taken, at least systematically, to train and instruct the mind. Discipline, in the military sense of the word, having in view the encouragement of good, as well as the punishment of bad conduct, was enforced; but it must be admitted that the latter mode generally predominated much over the former, necessarily, and from no proneness to severity in commanding officers. Had safe, and therefore salutary information been generally communicated in all fitting opportunities, it is not too much to affirm that the order of things would have been reversed—that punishment would have been little, and reward much. The bearing of these observations on the subject in hand, the health of seamen, is evident.

The most simple and comprehensive method for accomplishing that end, would have been the supply of amusing, and instructive books. Sailors can generally read, and many of them are fond of reading, as every one who has been much at sea, and observed the eagerness with which they fasten on any books which fall in their way, and read, either alone, or to a group of attentive listeners, knows. Yet, till a comparatively recent date, no measures were adopted, at least at the public expense, to gratify, and foster a taste, from which many advantages, direct and indirect, might have been derived, and may, and no doubt will, be derived. Less than 20 years ago, Bibles and Prayer-books, and, more lately, religious tracts, were issued gratuitously. The excellence of the measure, and of the motive which led to its introduction, are fully admitted; but it is no disparagement of either to say, that more was wanted. Knowledge must precede conviction; and, in the ordinary course of things, the mind must be opened and enlightened by ordinary means, before it can be made capable of understanding the doctrines, and receiving the benefits of religious truths.

Entertaining these opinions, which few, it is believed, will controvert, it is gratifying to know that means have been adopted to supply so great a want, and to obtain ends of such importance—agreeable occupation of the mind, and improvement of its faculties, leading to increase of health, and greater efficiency of national force. By an Admiralty order, dated August 1838, libraries are directed to be established in each of Her Majesty's ships, for the use of the crew, furnished at the public expense, and placed in charge of the schoolmaster. The books, amounting to 270 volumes for large, and 100 for smaller ships, exclusive of Bibles, are judiciously chosen, with the view of combining amusement, and instruction, and making the first subsidiary to the last. Besides the accomplished men now appointed to instruct the junior officers, it is further directed, by an order from the Admiralty, May 1837, that a fit person shall be appointed to give elementary education, comprehending reading, writing and arithmetic, to the sailor boys, and other seamen, and marines, who may require it.

There can be no doubt of the value of these measures, or of the beneficial results to which they will lead, if that on health alone is considered. Many less deserving have attracted much notice, and obtained high praise. Nowhere is there a mental field more capable, or more worthy of cultivation, than that which may be found on board ships; and it may be fairly lamented that it has been so long, so much neglected. Leisure, long absence from loved objects, and scarcity of external objects of interest at sea, dispose the mind to contemplation, rendering it highly susceptible of moral, and intellectual impressions, and, where it has the means, of being pleased and benefited by them. The time has passed when utter ignorance of every thing, but his immediate duty, with all the debasing, and destructive effects of savage ignorance, is thought essential to the character of a British seaman—implicit obedience, indomitable courage, and love of country. The time, too, has passed, when such ignorance, even if it were desirable, could be retained. Information of some kind will be communicated; it is therefore politic, if there were no higher object, that it should be of a sort to improve, not to deteriorate.

And it may be anticipated, when these, and other advantages, which are to be found in the Royal Navy, some of which have been alluded to above, are fully understood, that the service will become more popular than it has heretofore been. It is difficult to get rid of a bad character. Tradition, especially in seaport towns, and in merchant ships, is still rife of the evils, and of the sufferings, formerly endured in ships of war, some of them false, or exaggerated, but too many of them true. When there is neither personal evidence, nor convincing testimony, to the contrary, the easily prejudiced are too apt to believe that such evils, and sufferings are not yet banished from the public service; and it is therefore important to show, in how many respects, and to what extent, it has been really changed—how great is the preponderance of its benefits over its drawbacks. The best argument will be derived from experience. Good men, capable of thinking, and appreciating its advantages, and the number of such will increase, after knowing, and feeling them for some time, will not readily abandon them. They will speak of them to others, not yet sufficiently informed, or labouring under old prejudices; and thus the service will be sought, not avoided, and a ship of war, happy, healthy, abounding in present comfort, and prospective benefits, will be an object of desire to good seamen." xx.

All who have at heart not only the well-being of the Navy, but of their country, will respond to these sentiments, and applaud their spirit, and encourage their practical application. Whatever some may affect to think, the existence of this country as a free, to say nothing of an independent state, is bound up in the thorough efficiency of her marine, and the time has passed when the press-gang and the "cat" can make it powerful. The service must be rendered one which men will enter voluntarily, one where merit shall obtain rewards, and the mind shall not be debased, nor the body tortured. And, in our own department, much as has been done, something perhaps yet remains to do, and the assistant-surgeoncy should be of such a sort as the best young men who quit our schools should not be unwilling to accept.

*Ventilation.*—That by wind-sails is the only one generally adopted. But it is defective in many points. With strong breezes, in dry weather, a sufficient volume of pure air can be carried by them to, but cannot be sufficiently diffused along, the decks. They can reach only one point of one deck at a time; at that point the force of the air, in fresh breezes, is too strong, chilling, and often communicating disease to the persons on whom it directly falls.* Beyond that point it extends various distances, according to the force of the descending current, but does not extend far in most cases, and is often scarcely felt over considerable spaces between decks. Three wind-sails are generally employed, which are suspended from the rigging, pass down the hatchways, and terminate at any point between decks, which may most require ventilation. They vary in size, from eighteen inches to three feet in diameter; and when properly adjusted, so that their open, upper part is exactly opposed to the breeze, they transmit a sufficient supply of air, if it could be equally diffused over the interior of ships. Distribution is the difficulty. In some places, as has been stated, where the tubes terminate, there is often too much; in others there is, if any, too little. That is the greatest objection to ventilating by wind-sails. In calms it is of course unavailable, and in rainy weather cannot be practised.

The removal of stagnant air, the only efficient ventilation, cannot be obtained by wind-sails. It has been attempted by an apparatus contrived by Captain Warrington. It has sufficient power to draw air from any part of a ship, more indeed than is required, but that can easily be reduced. When foul air is taken from below, fresh air will descend from above to supply its place; and thus, it may be supposed, that both purposes will be answered,

---

* "It sometimes happens that men near the wind-sails, feeling discomfort from the chilling effects of their currents, tie them up during the night, and so, while undetected, which may be during a whole watch, deprive their shipmates of the fresh air which the wind-sails might supply, and which is particularly wanted by those at some distance from the hatches. It also sometimes happens that wind-sails, which are taken up during rain, are forgotten to be let down again when it ceases. These, though not necessary, are objections to this method of ventilating."

and that nothing more can be desired for ventilating ships. But two objections present themselves to the completeness of the process thus conducted. Both the ascending and descending currents are strong, too strong, if the ascending one were not otherwise deleterious, to be safely applied to the body in their direct course; and beyond the limits of their direct course, through the hatches, as happens with wind-sails, their influence will be little felt; at least it will not be equally and beneficially felt. It is therefore still imperfect. The Reporter observes that Captain Warrington's apparatus, though exposed to some of the objections which have been stated to wind-sails, has a great advantage over them; it will not, like them, be rendered inoperative, or useless by calms or rains. It can be worked in almost every kind of weather; and it might probably be made to accomplish the desired object by some contrivance like the following. Pure air might be drawn by it from an open port, or hatchway, and transmitted through tubes led along the sides of ships, the tubes being so perforated as to allow numerous small streams to pass from them to the centre of the ship. In this, or some such way, it appears likely that all which is wanted and desired from ventilation, might be effected, deficiency, and excess being equally avoided.

In small vessels, where the cooking apparatus is on the same deck as that on which the people are berthed, the heat of the fire, especially in cold weather, contributes to its ventilation.

### South American Station.

The first Report is on the squadron upon this station. The Report takes in each separate year from 1830 to 1836 inclusive. Each Annual Report is made up of four tables:—the first, shewing the total number of cases; the number of all diseases and injuries in classes; the number of cases sent to hospital, invalided and dead; with the ratio of each per 1,000 of mean strength. The second, shewing the total number of cases; the number of all diseases and injuries, in classes; the number invalided and dead: with the ratio of each per 1,000 of attacked. The third, shewing the number of principal diseases and injuries; the number sent to hospital, invalided and dead, with the ratio of each per 1,000 of mean strength. And the fourth, shewing the number of frequent, but not often fatal, diseases; the number sent to hospital, invalided and dead; with the ratio of each per 1,000 of mean strength.

We are informed by the Report, that the South American naval command embraces a great extent of coast and cruizing ground. It extends, on the east side of the peninsula continent, from Para to Cape Horn, and on the west from Cape Horn to Panama, and thence to California. It reaches from the 30th degree of north, to the 58th degree of south latitude across the Pacific, from the equator to 58° south, across the South Atlantic; and from Cape St. Roque in the 35th, to California in the 120th, degree of west longitude. It is therefore exposed to almost all degrees of atmospheric temperature, from the highest to the lowest, with the ordinary meteoric agencies thence arising. Many of the ports and places of anchorage, and adjacent territories, differ from each other in almost every manner and degree. In relative position, and nature and extent of exposure, in respect of contiguous land, its general form and distance, and influence on wind and rain, and in the soil, and products of the soil, there is great, sometimes extreme difference, in the various places resorted to by British ships. The places principally frequented are Rio de Janeiro, Buenos Ayres, Bahia, Pernambuco, and Para, Valparaiso, Callao, Coquimbo, Panama, and San Blas. In some of them art has been busy and effective; large towns have been built, and the ground has been cleared and cultivated in the neighbourhood of the towns, and on the margins of the bays and harbours; in others, beyond rearing houses and stores, things remain very nearly in their natural condition. Hence extensive marshes are in close contact with many of them; while in others, but more from natural formation than the labours of man, dry land, and little productive of vegetable matter, abounds. The places named are, with three exceptions, situate within the tropics, some near their external limits, some close to the equator, and others at different intermediate points; yet with all such difference in position, soil, products of soil, and climatorial heat, the inhabitants of the shores of this vast continent whether permanent or occasional, enjoy a high and a singularly uniform degree of health.

Epidemic diseases are scarcely known, they certainly are not destructive. The fever which frequently makes such havoc in the West Indies, never makes its appearance here. To such fevers as those which often are so fatal in Africa and Asia, in North America, and even in the Mediterranean, the people of this continent are not subject. They are not, more than the inhabitants of every other portion of the globe, free from febrile disease, but they suffer little from it. Even the malignant cholera has not yet, it is supposed, reached South America.

"Why is it," asks the Reporter, "that in a land-locked harbour, in this part of the world, under a powerful sun, surrounded by marshes and rank vegetation, ships lie for months, or years, without the occurrence of a single case of concentrated fever; while in other places, in Africa, in Asia, in North America, and more especially in the West Indian islands, things, to superficial observation, which appear to be the same, are productive of so much disease and death?

The only thing in the shape of a reason which has been attempted is altogether incapable of satisfying an inquisitive mind. It has been alleged that exposure of the coast to the wide ocean, and the cooling influence of the trade winds, reduce the temperature so much, as to take away from other agents the power of exciting the more fatal forms of intertropical fever. To such unsatisfactory reasoning the thermometer offers instant and positive refutation. From Para to Rio de Janeiro the temperature through the year, and during corresponding periods, is higher than in that part of the Gulph of Mexico which stretches from Vera Cruz to Havanna. In the latter, fever is a frequent and fatal malady; in the former it is of rare occurrence, and has little force or fatality when it occurs. If high degrees of heat alone, or acting on swamps, and on abundance of vegetable and animal matter, could occasion such fever as that which proves highly destructive in the West Indies, it is clear that many parts of South America could not only not escape, but must suffer severly from it. Other causes, then, if we are to come to the truth, must be sought."

The Reporter recommends, and the idea is most judicious, a scientific survey of the soil, subsoil, and other permanent parts of the structure of the locality where it exists, in connexion with temperature, resulting atmosphere, and natural products.

We trust that the medical officers of both the navy and the army, and the civil practitioners abroad will devote their attention to this subject, and supply what is felt as a desideratum of the first importance. The Reporter anticipates, and we are inclined to go with him, more good from investigations of this sort, than from quarantine enactments or a sanitory cordon.

It is peculiar to this command, that, during peace, the ships are employed altogether on an alien coast. Along its whole extent, and nowhere within the limits of the command, excepting the recent small settlement at the Falkland Island, is there any British possession. Hence there are no hospitals for the reception of British sailors; the want of which, though it may not materially and directly affect life, increases the necessity for invaliding.

*Year* 1830.—The average numerical force during the year was 2,933. There were 28 ships and vessels of all descriptions, of which one was of the line, six were frigates, and the remainder sloops. The number of men appears small for the number of ships; but of the 28 employed, 12 were packets, and one was a vessel on passage to India. The packets have very small crews, and, being on the station during short periods, seldom send more than one quarterly report each from it. For each quarterly report, one-fourth of the complement of the vessel is allowed in calculating the mean force of the year; and hence the apparent want of proper proportion between the number of ships and of men employed.

The total number of sick and hurt was 3,611, being at the rate of 1231·2 per 1,000 of mean strength—a large proportion certainly, but not extraordinary and not excessive, when all the circumstances which swell the sick lists in the naval service are considered. Out of the whole number of sick and hurt, 27 died, or were killed by accidental violence, being at the rate of 9·5 mortality of mean strength, and 7·8 per 1,000 to the number of sick and hurt. There were 76 cases of invaliding, being at the rate of 25·9 per 1,000 of mean force. The number is considerable, absolutely and comparatively, but is much augmented, there is little doubt, as has been noticed above, by the absence of hospitals.

There were 236 cases of fever and febrile disorder, divided into 193 of continued, 31 of remittent and 12 of intermittent fever. Independent of detailed accounts, it may be inferred, that in most instances the diseases thus classed were slight and simple, since out of the whole number 227 were cured in ships; there were only six fatal terminations, and two, from resulting debility were invalided. The eight cases not terminating in complete recovery, were among the continued fevers. In no ship was there any prevalence, or any thing peculiar, either in the origin or nature of the febrile disorders.

The order "inflammation with fever," or that of primary organic inflammation, was, as usual, numerous, amounting to 1,035 cases, four of which terminated fatally, and nine were invalided. Half, however, of the whole number were cases of slight cutaneous inflammation of the lower extremities, frequently terminating in some form of suppuration.

There were 169 cases of rheumatism. Passages round Cape Horn to and from the hot and temperate portions of the station, may be expected to excite the disease. It was, however, more tractable than it often is, all the affected, excepting three cases invalided, being cured on the station. But probably the name of "rheumatism," has been abused, or the complaint often feigned.

There were 53 cases of inflammation of the lungs, and of their membranes and air-passages; all of them, with two exceptions, one of death, and one of invaliding, were cured on board the ships in which they occurred.

There were only seven cases of phthisis, and six of hæmoptysis; of the former, three terminated in death, and two were invalided; and of the latter, three were invalided. The proportion of consumptive disease is therefore not large. The ascertained loss from all diseases of the respiratory organs, acute and chronic, is only four: yet if all the persons invalided for phthisis eventually died, independently of those invalided for hæmoptysis, the mortality from pulmonic disease would be greater than that from fever.

There were 63 cases of dysentery, two of which terminated fatally, and one was invalided. Of other bowel complaits, diarrhœa, colic and common cholera, there were 418 cases; but, with the exception of five invalided, they were all cured on board.

The catarrhal cases amounted to 337. In many instances there was a good deal of febrile and respiratory disturbance, having much of the character of catarrhal fever; but they were all cured on board.

Of delirium tremens only one case in an *officer*! How this contrasts with what happens in the army.

There were 202 cases of venereal disease; viz, 118 of syphilis, and 84 of gonorrhœa. A large proportion of the syphilitic affections were contracted in the South Sea, principally at Otaheite, and neighbouring islands. There was something peculiar in the disease there; often without any appearance of chancre or appreciable lesion in the generative organs, syphilitic disease was developed in the groins; one case of syphilis proved fatal, chiefly by hæmorrhage from the anus.

Ulcerative cases were numerous, amounting to 335; of which, 20 required to be invalided. "Ulcer is a prevalent, generally the most prevalent, form of diseased action on the station. Few ships, if they are much in harbour especially, escape suffering from it to some extent, and that mostly in the hottest seasons. On first arriving from England, it generally assumes an acute character, makes rapid progress, involves the cellular tissue rather extensively, often becomes phagedenic, or sloughs, and is attended by much febrile disturbance. The resident inhabitants are prone to similar forms of disease, to various affections of the skin, and subcutaneous structure, and of the lymphatic system; the negroes, more especially, to elephantiasis, and some modifications of lepra. For these diseases there is evidently an endemic cause, connected, no doubt, with atmospheric heat, but not dependent entirely upon it.

Out of 335 cases of ulcer, the large proportion of 146 were in the Warspite. That ship—a ship of the line, and carrying the flag of the commander-in-chief—had, at the end of the year 1830, been 18 months without change of position in the harbour of Rio de Janeiro ; she was, therefore, constantly exposed to the endemic cause of the disease prevailing there during a long period. Sometime after her arrival from England, the ulcerative diathesis began to declare itself. Slight injuries, musquito-bites, and other trivial lesions of the surface, were followed by diffusive inflammation terminating frequently in ulcer. The ulcer manifested sometimes diminution, but much more frequently violent increase, at the onset, of vascular action ; occasionally it became indolent soon after its formation, but in most instances retained long a dangerous degree of activity. As the season advanced, and the weather became so hot that the thermometer seldom fell below 80°, the diathesis became stronger and more general. Often, without the reception of any external injury, a pustular point, with high surrounding inflammation, most commonly near the knee or ankle-joint, was exhibited. Within a few hours from the first appearance of the affection, the inflamed circle was much extended, more tumid, and intensely hot, red and painful. From the edges of the pustular central point, which discharged, or rather yielded, on pressure, a small quantity of pus, phagedenic action commenced, and often extended with astonishing rapidity; there were frequently extensive sloughs, involving large portions of the cellular tissue, before the destructive action could be arrested. In all such cases there was much febrile disturbance ; indeed, the disease might, with propriety, have been designated ulcerative fever. The treatment included and consisted chiefly in the application of general remedies. Till the destructive violence of action was subdued, reductive measures, especially drawing blood, both from the system and the inflamed parts, were freely adopted. The diathesis and development were the very reverse of the scorbutic, and an opposite line of treatment was pursued. Here they were clearly and closely connected with plethora and augmentation of vascular action. The disease was almost entirely confined to new men—recently recruited marines, and sailor-boys, many of them embarked for the first time. In many instances the subjects had passed suddenly from a state of complete indigence, under-feeding, and irregularity, to full and excellent rations, and to a life of order and of ease." Nothing could be discovered in the condition of the ship to give rise to the affection, that seemed attributable to her long continuance in harbour, and consequent long-continued exposure to its influence, in connexion with the peculiar susceptibility of a portion of her crew.

*Year* 1831.—We shall content ourselves with merely pointing out the differential characters of each year, having given so fully the prevailing disorders on the command.

The ratio of mortality in 1831, was almost exactly the same as in the preceding year, being under one per cent. Though the mortality was at the same rate nearly during the two years, there was considerable difference, on comparing them, in the causes of mortality. Thus, in 1831, there was less loss from fever than in 1830, while there was more from bowel complaints and accidents. In neither was the loss, from any source, considerable, excepting from that of accidents in 1831 ; nor was there any formidable or prevailing form of disease.

The mean numerical strength of the year was 2,521. The whole number of deaths during 1831 was 23, but six of them were the results of falls from aloft, and other accidental causes, reducing the mortality, from disease, to 17, and the rate of death to life, the effect of climate, and the ordinary agencies of nature, to 6·7 per 1,000. The squadron suffered very considerably, however, from the number it was thought expedient to invalid. No fewer than 89 persons were thus disposed of, being at the rate of 35·3 per 1,000 invalided of the number employed. A large proportion of the cases were chronic inflammations, or their results; many of which might have been saved to the command, perhaps to the service, had there been hospitals for their reception on the spot; for it cannot be doubted that, in many instances, the time necessary to reach an English hospital will so aggravate disease as to render it mortal, which, eight or ten weeks before, might have been arrested, and eventually cured, under efficient treatment; that is, had the surgeon been able to add to a proper system of therapeutics, the room, the rest, the air, and the rightly ordered and rigidly enforced dietary, which a well constituted and well conducted hospital supplies.

There was only one ascertained death from all the diseases of the respiratory organs.

The number of diseases of the liver denominated inflammatory, whether acute or chronic, was proportionately larger than common, namely, 65; of which it was necessary to invalid nine. A large proportion of them, viz. 26, were in the Lightning. The ship was employed in the bay of Cape Frio, in retrieving treasure and stores sunk there by the wreck of the Thetis. The ship's company suffered considerable fatigue, and were much exposed to variety of weather, in working diving-bells, and other operations arising out of that undertaking. They could not escape the influence of miasmatal exhalation, and sanatory orders could not be so efficiently carried into effect, as under ordinary circumstances, and modes of life of a man-of-war. All the cases, with the exception of one invalided, were cured on board.

There was a rather large proportion of primary venereal affections, viz. 156; of which 101 were syphilis, and 55 gonorrhœa. Though the former were frequently tedious, they were all, with two exceptions invalided, finally cured on board. The ratio of syphilitic cases is high, particularly as compared with the gonorrhœal; a great majority of the former occurred in the Pacific. There is a striking difference in the frequency, as well as in the force of the venereal, especially the syphilitic form of the disease, on the two sides of South America; on the Eastern or Atlantic side, it is comparatively a rare and mild disease; while in the Pacific it is frequent, and often difficult to deal with, occasionally exhibiting peculiar features, and offering much resistance to treatment. In illustration of difference, as to frequency, it may be stated, that in the Warspite, during seven months, there was only one case of syphilis at Rio Janeiro; while in the Seringapatam, employed during the year in the Pacific, there were 26 cases. The former ship had a complement of 588, and lay all the time in the harbour, the ship's company having free intercourse with the shore; the latter had a complement of 300 and was a good deal at sea. Such difference is common, and difficult to explain; neither climate, at least in temperature, nor modes of life, as far as they are known, will account for it.

Ulcer was as frequent as ever, but less severe. In the Warspite it had lost much of its violence, and was less frequent. Out of 314, the number of cases in the squadron, it was found necessary to invalid no more than eight; whereas in the former year, that measure was required in twenty cases.

*Year* 1832.—"The squadron enjoyed a very high degree of health during the year 1832, as compared with other portions of the service, with the civil inhabitants of places celebrated for salubrity, and even with itself, high as it then was during the two immediately preceding years. The ratio of mortality for the year, and having reference to every instance of death, whether from disease or accident, was 6.2 per 1,000. When it is considered that the greater portion of the command is intertropical, and that the ships' companies were often, some of them constantly, exposed to high degrees of atmospheric heat, the trifling loss sustained becomes more striking. The fact is interesting in every point of view, but especially as it shows, in connexion with many others which might be adduced, that simple elevation of temperature, however great, has little, if any, power in the subversion of health; or at least, that it does not occasion epidemic forms of disease, suddenly and extensively destroying life. The entire health-history of this part of the world leads to that conclusion. Tables like the present, could they be obtained through a long series of years, would confirm it. In connexion with the subject, it may be stated, that the Warspite, with an average complement of 600, lay the whole year in Rio de Janeiro harbour, and did not lose a man, and had only seven cases of fever, viz. two of intermittent, four of gastric remittent, and one catarrhal."

There were six deaths from fever, two from continued fever, and four from remittent. The latter happened, nearly at the same time, in the Pylades. The case is worth noticing. A party of men were detached from the ship to the Island of Macenasa, where a merchant vessel had been wrecked, and where some of the party from the Pylades remained 28 days. The island is 25 miles north of Pernambuco, close to the main land, which is high, the island itself being low, flat and marshy. The men employed on that service, saving property from the wreck, had hard work, and were exposed alternately to a powerful sun and to heavy rains. Some of them slept on the wreck, others in tents on the island. New rum was cheap and accessible, and absolute sobriety could not be preserved. Eighteen days after the commencement of those operations, fever broke out, and extended to 21 persons, four of whom, as has been stated, died. The fever was confined to the men employed about the wreck; though the Pylades lay twelve days close to the place, no one not so employed was affected; while the half of all those employed, were attacked. The Reporter cites this as illustrative of the importance of avoiding the predisposing causes of fever. The Pylades, herself, lay twelve days near its source, no one who continued to live and sleep aboard was affected by it. But with the shore-party, fatigue, long-continued exposure to the sun and to rain co-operating, irregularity in eating and drinking, abuse of spirits, and other excesses, were in full operation. Besides the men thus rendered susceptible were more directly exposed to the essential cause of the disease, from sleeping on the island, or on the wreck close to it.

*Year* 1833.—Though the rate of mortality is higher in 1833, than it was in 1832, it is yet low. It is lower than it was in 1830 and 1831, in neither of which did it reach one per cent. and is, compared with many other divisions of the world, whether in military or civil life, inconsiderable. From all causes, internal and external, there were 18 instances of death, being in the proportion of eight persons dead out of every 1,000 persons employed. The causes of death were inflammation affecting various organs, consumption, fluxes, and accidents; their relative force being in the order in which they are stated. There were eight fatal terminations from inflammation, four from consumption, three from affections of the bowels, and three from falls or other accidents. It seldom happens in so large and extended a squadron, during so long a period, that the causes of death are so few in number, whatever combined force they may possess.

The mean force of the year was 2,217. The entire number of sick and hurt was 2,848, being at the rate of 1284·6 per 1,000; there were 18 fatal cases, being at the rate of 8·1 per 1,000; and 45 were invalided for change of climate, or having become unserviceable, being at the rate of 20·3 per 1,000. The ratio of sick and hurt, and of dead, is higher, while that of invalided is lower, than during last year. The proportions of sick and hurt, of dead, and of invalided, are less than they were in 1831; but while the proportions of dead, and of invalided, are less, that of sick and hurt is more than in 1830. The rate of invaliding is generally high in the naval service, especially, and, as has been stated before, in consequence of want of hospital accommodation in this command; during the present year, taking that circumstance into account, it is, being as nearly as possible, two per cent. moderate.

Of 18 deaths, which occurred in the year, three were from accidental causes, and seven, or almost half the entire mortality, from disease, were from inflammatory affections; as was a large proportion, namely, a fourth of the invaliding. Of these seven cases, two were from inflammation of liver, one of stomach, one of bowels, one of lungs, one of brain, and one rheumatic. Four of them were in the Spartiate, a ship of the line, not long from Europe, after being employed during winter in the North Sea. The sudden transition from low to high temperature, will sufficiently account for the frequency of such disease in her; 261 of all the cases in this order occurred in her, in six of which invaliding was resorted to; so that there was a reduction of 10 in her complement by inflammatory diseases.

There were 98 cases of primary venereal disease, 66 of syphilis, and 32 of gonorrhœa. As compared with last year, there is considerable diminution, as there is when that year is compared with 1831, and 1831 with 1830; there being in the first year of the series, viz. 1830, 202 cases; in the second, 156; and in the third, 119 cases. The mean force of the respective years in the same series being 2,933, 2,521, 2,579 and 2,217. The reason of the diminution is not evident. Yet there was a similar diminution in the amount of simple ulcers, compared with the last and two preceding years. In this, as in the specific venereal ulcer, there has been progressive, reduction not only in frequency, but also in force, during four consecutive years, commencing with 1830, and nearly at the same numerical rate. Thus, in the first year there were 335 cases, in the second 314, in the third 222, and in this, the fourth, 162. The coincidence is curious.

"An interesting question connected with the vital statistics of the navy, is that of the influence of sea and harbour duty relatively on the health of ships' companies. It is, however, as has been already stated, one difficult, if it be possible, as ships are generally employed, to answer satisfactorily. They are seldom more than a few weeks at sea without communicating with the land, and remaining during longer or shorter periods in harbour. On going again to sea, either to cruize, or for passage, if disease should not prevail at the time, but break out within a moderate time afterwards, it may be doubtful whether such disease is to be ascribed to any thing in the harbour the ship has left, the place where she is, or in the ship herself. There can be no doubt, putting aside the causes of many pestilential diseases derived from certain harbours, that higher discipline, more regular modes of living, and more temperate habits can be otained at sea than in harbour, and that so far the balance is in favour of cruising. But supposing that the harbour resorted to is not charged with any detrimental miasmata, a sea-life has many countervailing disadvantages, whatever their comparative amount may be. Want of fresh provisions, necessary exposure to all kinds of weather, imperfect ventilation from the necessity of shutting ports &c., lack of amusement, and a monotonous manner of life, after a while impair vigour, and cause or lead the way to many maladies. The fleet blockading the ports in the Mediterranean toward the close of last war, enjoyed a high degree of health, though long at sea; but that fleet united many of the advantages of sea and harbour duty life. It had the high order and regularity of the former with the fresh provisions of the latter, supplied by transport ships sent alongside. It had the excitement of a stirring though uniform occupation, and the hope of encountering the enemy. At sea the danger arising from the poisonous exhalations of some shores are avoided; beyond that it is not easy to determine whether, and to what extent, a life at sea is more salubrious than in port. In relation to this matter, as shewing that mere continuance in port is not prejudicial to health, a few particulars regarding the Warspite may be stated.

That ship, during the last term of service in the South Atlantic, viz. from June 1829 till January 1833, lay upwards of three years in the harbour of Rio de Janeiro. She was anchored there at first two years without change of place, was then absent five months at the Cape of Good Hope and Isle of France, when she returned thence to the anchorage at Rio, where she remained upwards of another year before proceeding to England. During the whole period, the ship's company enjoyed a high degree of health. During the last three years, the total number of deaths was eight, of which four were the effects of accidents, four only resulting from disease, viz. one from fever, one from consumption, one from tetanus, and one from diseased spleen. The ship's company varied a good deal in number, on account of the frequent reception and discharge of supernumeraries, but the average was upwards of 600; the rate of mortality from all diseases being about two, and from all causes about four in the 1,000. There was no ship on the station of the same force, and much at sea, with which a comparison could be instituted; even if the objections to the validity of such comparisons alluded to above, could be obviated; but, by whatever measure tried, a higher degree of health than that enjoyed in the Warspite cannot well be supposed."

*Year* 1834.—The rate of mortality was a little higher in 1834 than in 1833, higher by a third than in 1832, nearly the same as in 1831, and the same as in 1830. These two years, the first and the last yet examined, though the least healthy of the series, were yet healthy, the rate of mortality being something less than one per cent. Part of the small increase of this year, as compared with last, depends upon increase of febrile power, and part upon an unusual proportion of fatal affections of the brain, resulting from what may justly be considered accidents, though not external accidents, that is, from causes which were not necessary, and might have been avoided.

The mean force of the year was 2,231, being only 14 more than that of last year. The total number of sick and hurt was 3,403, being in the ratio of more than 1500 per 1,000, the highest ratio of sick and hurt yet encountered on this station. But though the ratio of sickness is higher than in any of the four preceding years, some of the resulting ratios are equal, some lower. Thus the ratio of mortality is 9·5, the same as in 1830, the total number of deaths this year being 21.* The ratio of invaliding is lower than it was in 1830, much lower than in 1831, and almost exactly the same as in 1832; this year 53 were invalided, being at the rate of 23·8 per 1,000.

Inflammatory affections were numerous, even when compared with former years, on this station, and with other stations; 914 cases were under treatment, being upwards of 40 per cent. of numerical strength; so that almost every second man suffered from some form of inflammation. But though the number attacked was greater than last year by about six per cent. the loss by death was much less, while that by invaliding was not much greater. This year there were four deaths, being less than two deaths out of 1,000, while last year the proportion was more than three per 1,000.

There were 134 cases of inflammation of the lungs and their membranes, though only one death, and four cases of invaliding. The affections of the membranes, particularly those lining the air-passages and cells, were more numerous than of the substance of the lungs. Of the former there were 72 cases, of which 71 were in the Spartiate. In that ship there were, of all inflammatory affections of the respiratory organs, 103 cases, about three-fourths of all in the squadron. In the harbour of Rio de Janeiro there is great general tendency to bronchial disease, though varying much at different periods; this year it was very prevalent.

Out of 82 cases of disease of the brain, four terminated fatally. One was apoplexy—three were "drunkenness."

Primary venereal affections were a little more numerous, and there was a considerable increase in simple ulcers.

*Year* 1835.—The rate of mortality is still lower during the year 1835 than it was during any of the five preceding years. It is strikingly low, however estimated, compared with other places and classes of persons, being that of one death out of every 200 employed nearly. It is difficult to account for such paucity of fatal result, when compared even with the results of former years on the same station. There was little difference in the nature of the service, or distribution of force—none from which such results could be anticipated; and the wreck of the Challenger, with long consequent exposure of the people, and much privation and hardship, was a disaster, from which increase of sickness and mortality was to be apprehended.

The mean force of the year was 2,622, an increase of nearly 400 on that of last year. The total number of sick and hurt amounted to 3,577, being at the rate of 1364·2 per 1,000; 14 of which terminated in death on the station, and 73 were invalided. The ratio of deaths, one of which was from accident, is 5·3 per 1,000.

---

* "A fatal case from atrophy, in the Beagle, was omitted; the return could not, at the time, be found."

The ratio of invalided was 27·9 per 1,000, which is higher than that of last year, or of any preceding year, except 1831. It is remarkable, however, that eight were invalided for epilepsy, four from one ship. There was probably malingering here.

There was a notable decrease of inflammatory affections of the lungs, but an increase of phthisis.

*Year* 1836.—There is little in the Tables of this year to elicit remark; they have much similarity to those of the six preceding years. The ratio of mortality is not so high, nor so low, as in some of the foregoing; it is about equal to the average of the seven years of which it is the last. The highest was 9·5 per 1,000, occurring in two years, 1832 and 1834; the lowest was 5·3 per 1,000, that of 1835; this year it is 7·4 per 1,000. The same remark applies to the ratio of sick and hurt and of invalided, with this qualification, that the ratio of the latter is higher than that of any other year, excepting 1832, than which it is very little lower.

There were 69 cases of invaliding, being in the ratio of 32·1 per 1,000. The ratio is high, comparing it with most years on the station, and with other stations. The great majority of invalided cases were from inflammation; cutaneous diseases, including scrofula and syphilis; spasmodic disease, including epilepsy and ulcers; and accidents, exclusive of ruptures, of which, however, there was an increased number.

The Reporter presents us with a Table for seven years, from 1830 to 1836, shewing the total number of cases; the number of all diseases and injuries, in classes; the number of cases sent to hospital, invalided and dead; with the ratio of each per 1,000 of mean strength.

This Table shows that the annual rate of mortality, on an average of seven years, in the South American command, was, from all causes, 8·9 per 1,000; from disease, distinguished from external causes, 7·7 per 1,000 of force. It should be observed, that the ratio refers not only to persons dying in the command, but also to those who died afterwards from disease, which originated, or appeared to originate, there; for the invalids sent to England have been carefully traced, and as many of them as died in home hospitals have been added to the mortality abroad, and included in the sum total from which it is deduced. Thus, it is presumed, a complete view of the influence of the climate is given.

The aggregate numerical force of the seven years was 17,254, which was reduced by death to the extent of 136 only on the station, and 19 in home hospitals, making a total loss of 155 by death. But of 136 fatal cases which happened on the station, 21 were the result of external injuries, falls from aloft, and other accidental causes, bringing down the number of deaths from disease within the limits of the command to 115; so that the average annual loss by death, the effects of climate, and the ordinary agencies affecting life, was no more that 6·5 per 1,000 of the number employed in South America. This, or taking the more just and comprehensive view which includes the deaths from, as well as those on, the station, and which makes 7·7 per 1,000, is a singularly low rate of mortality; it is much under the average of the United Kingdom in persons of corresponding ages.

The Reporter regrets the impossibility of comparing the mortality in the

British squadron with that of the French or United States' squadron, or with that of the inhabitants of the sea-port towns of South America. He remarks that—there has long been little doubt respecting the salubrity of the climate of South America, at least of that large portion which stretches from the equator on the east, south, west and north, over the whole of its remaining extent. Experience had proved the remarkable immunity which it enjoyed from such epidemics as those which often devastate the West Indies, the tropical shores of Africa, or as are sometimes severely felt in the East Indies. But a position may be exempt from such violent visitations of disease and sources of mortality, and may yet not be highly favourable to health, as this is now satisfactorily shown to be. When in connexion with the vast extent of the continent, it is considered that a great portion of it lies within the tropics, that most of it is still in a state of nature, almost all of it thickly covered with vegetable and animal matter, in vigorous growth, or rapid decomposition, and that creeks, lagoons, and marshes, are in many places abundant, its freedom from violent epidemics, independent of its general healthiness, is indeed remarkable. No satisfactory explanation can be offered.

Altogether 485 persons were invalided during the seven years ; of which 466 were on the station, and 19 in home hospitals belonging to the station. The ratio per 1,000 of the whole number invalided, on account of disease exhibiting itself on the station, or of injuries received there, is 28 per annum during the seven years.

A second Table shews the total number of cases (from 1830 to 1836) ; the number of all diseases and injuries, in classes; the number invalided and dead ; with the ratio of each per 1,000 of attacked.

The aggregate force of the seven years being 17,254, the first striking fact established by the Table is, that the mortality from fever was in the ratio of 1·3 per 1,000 per annum of the number employed, though the number treated annually was in the ratio of 115 per 1,000 of force, the whole number treated being 1,984. Probably, however, many of these were cases of symptomatic fever.

Under the head of consumption, 55 cases were treated ; of which 15 terminated fatally on the station, and 10 in home hospitals, out of 14 transmitted from the station, making a total of 25 fatal cases; the annual rate of mortality from the disease was, therefore, 1·5 per 1,000 of the employed. Seeing that 30 of the 55 persons attacked were cured, it may be inferred that all the cases thus classed were not cases of true consumption; that though they simulated, they did not all really possess the true tuberculous character. Though the mortality from consumption is so little, it is, including the deaths in home hospitals, more than from any other disease in the command.

From all diseases of the lungs, exclusive of one from catarrh and one from bronchitis, which probably should have been added to the consumptive, two from hydrothorax, and one from dyspnœa, there were 35 deaths; being at the rate of something more than two per 1,000 per annum mortality from this division of disease.

There were 282 cases of disease of the liver, designated "inflammation," of which seven terminated fatally, six on the station and one in home hospital. The annual rate of deaths is 1·20 per 3,000, which, considering the prevalent high temperature of the station, is low. Neither, keeping that agency in view, was the loss to the command by invaliding, from this disease, great, the whole number thus disposed of being 37, a little more than two per 1,000 per annum of force.

There were 373 cases of dysentery; of which 17 terminated fatally, 16 on the station and one in home hospital, and 13 were invalided. The annual rate of mortality from the disease is, as nearly as possible, one per 1,000. After consumption and fever, this was the most fatal form of disease in the command.

There were only ten cases of delirium tremens, occasioning two deaths in the seven years.

Thus, says the Reporter, there were 125 deaths from principal diseases and accidents. There were, besides, two suddenly fatal cases designated asphyxia, one of palsy, one of diseased heart, one of cynanche, one of erysipelas, four of dropsy, one of scirrhus, one of syncope, one of bronchitis, one of sun-stroke, one of insanity, one of inflammation of the heart, one of hydrocephalus, one of jaundice, one of catarrh, four of rheumatism, one of dyspnœa, two of abscess, one of diseased spine, and three of disease, in the returns, designated drunkenness; making, in addition to the deaths from classified diseases, 30 fatal cases, and giving, with them, the total 155 cases of death on and from the station in seven years. The Reporter comments, in conclusion, on the infrequency of organic obstructive disease, such as jaundice and ascites, as evidence of the salubrity of the South American climate.

Another Table shews the total number of frequent, but not often fatal, diseases; the number sent to hospital, invalided and dead; with the ratio of each per 1,000 of mean strength.

These diseases are—superficial inflammation of the extremities—rheumatism—catarrh—and diarrhœa.

In respect, says the Reporter, of the first, the superficial inflammation of the extremities, there is no doubt that the nature of some parts of duty, especially that of cleaning decks by washing, and stoning, operates extensively; but then there is little doubt that some climates co-operate with the duty, and aggravate the evil. The inflammation in question is generally confined to the lower extremities, and to the lower part of them, seldom reaching above the knees. It often terminates in abscess, which, though seldom involving more than the skin and contiguous cellular tissue, sometimes extends deeply and widely, and proves tedious of cure. Such results, with the frequency of the disease, render it a source of considerable suffering, and temporary reduction of strength. The number of cases treated being 2,879 in seven years, the ratio per 1,000 of force is 166·9 per annum.

The annual ratio of rheumatic cases was 72·3 per 1,000 per annum of force, the total number treated being 1,248, of which 43 were invalided, and four, probably from attacking internal organs, terminated fatally. The annual ratio of invalided is 2·5 per 1,000 per annum of force, which, considering the nature of the service, the character of the disease, the difficulty which is sometimes encountered in detecting imposture, when it is feigned, that the name is sometimes applied to other affections, and the want of hospitals, is not high.

The ratio of attacked by catarrh is 139·8 per 1,000 per annum of force;

25 were invalided; being in the ratio of 1·5 per 1,000 per annum. The ratio of attacked is moderate, but that of invalided is high. One out of the whole number terminated fatally.

The ratio of cases of diarrhœa is 80·6 per 1,000 per annum of force; of which seven terminated in a chronic condition, which led to invaliding: none terminated in death.

A voluminous Appendix presents the details on which the preceding Tables and Calculations were based. We take our leave of the South American Station with any thing but a low opinion of its healthiness. The facts which have been stated in regard to it shew how utterly unfounded are many of our notions on the prejudicial influence of high temperatures.

### II. WEST-INDIES AND NORTH AMERICA.

*Year* 1830.—Tables of the character already alluded to in the Report on the South American Squadron usher in the observations and inferences of the Reporter. He remarks that—to ascertain the influence of climate on health in the most satisfactory manner, it would be desirable to consider that of the West Indies, and of North America separately. It would lead to more correct conclusions to have a set of Tables indicating the ratio of sickness, invaliding and death in each, the Bahama Islands being classed with the West Indies. The Bermudas form a kind of neutral ground, a connecting link between the West Indies and British North America, possessing some of the features of each, but not generally having the well-marked character of either. Their appreciable climate and morbid manifestations, however, are more West Indian than North American, and it would therefore be right, if not taken by themselves, to group them with the former, rather than the latter. But the whole being under one commander-in-chief, and the vessels continually changing places, passing from intratropical to extratropical latitudes, and *vice versâ*, such Tables cannot be constructed.

The Reporter examines the question whether this disposition of the Naval Force is, on the whole, conducive to the health of seamen, and without deciding it either way, he concludes that the changes of climate augment inflammatory diseases though they diminish fevers.

The mean annual force amounted to no more than 3,326. The total number of sick and hurt was 5,070, a very large proportion, being at the rate of 1524·3 per 1,000. Of all the sick and hurt, 313 were sent to foreign hospitals, being at the rate of 9·5 per 1,000 nearly, likewise a large proportion. From various ships 138 were invalided, being at the rate of 41·4 per 1,000, and 40 were invalided from hospitals, making a total of 178 invalided during the year, and being at the rate of 55·3 per 1,000 of mean strength. Out of so large a number of sick, however, only 21 died on board ships, yielding the very low ratio of 6·3 per 1,000; but 54 of hospital cases terminated fatally, making a total of 75 deaths; the ratio of the total of mortality of mean annual force being 22·5 per 1,000. The numbers of hospital establishments within the limits of the station, and the consequent facilities of sending sick to them, with the practice of sending every person attacked by fever at Port Royal at once to hospital, will account for the relatively small number of deaths at sea.

The loss, continues the Reporter, to the service resulting from death, whether at sea or in hospital, was not great. In most tropical positions it would not be considered severe, and is any thing but formidable, the West Indies being a large portion of the field of service; but the loss to the squadron from invaliding was certainly considerable, and deserves consideration.

The most remarkable circumstance, on examining the invaliding column, is the small number found in it resulting from essential fever, and the proportionably large from the pure forms of inflammatory disease, viz. five from the first, and 54 from the last, on board ships. It is the more remarkable as regards fever, considering the number of cases of periodic fever found in the medical returns, which so frequently require, sooner or later, to be invalided; and it cannot be doubted that the smallness of the number on this occasion depended, in part at least, on the favourable change of climate, from hot to temperate or cold. But that very change would tend to increase the number of inflammatory attacks, and of those invalided on account of them.

The number of cases of idiopathic fever amounted to 759; many of them, however, were so slight and transient as to be designated ephemeral. The whole are divided, according to the medical returns into 586 of continued, 84 of remittent, and 89 of intermittent, fever; of the first, 86 were sent to foreign hospitals, four were invalided, and five ended in death on board: of the second, 48 were sent to hospital, and two terminated fatally on board; and of the last, eight were sent to hospital, and one terminated fatally on board. It is curious that out of all the cases of periodic fever so often requiring invaliding, not one was so disposed of; while four of the continued cases, where perfect recovery, when it takes place, is so much more common, were sent to England for change of climate.

The following statement bears on a curious coincidence, or fact.

"The Blossom (surveying ship) suffered more than any other vessel from a fever designated remittent. She had 76 cases, 46 of which were sent to hospital, where 10 terminated in death, and two deaths occurred on board. The disease broke out, and ran its course, in a few weeks, off Belize, a position abounding in the cause of periodic fever, two days after the ship's arrival there from the anchorage at Port Royal. There is difficulty, as there generally is, in tracing this fever, which appears to have been the true endemic West Indian fever, not ordinary remittent, to its source. No fever of the kind existed at Belize, or other point in the Bay of Honduras, at the time it broke out in the Blossom, nor does any appear to have occurred during its course. The necessary conclusion therefore is, that the cause of the disease was either generated in the ship, being an intrinsic product of herself, or was derived from an extrinsic source, at some place, where she had previously been, having lain so long dormant in the persons of those afterwards affected. The surgeon of the ship inclines to the former opinion, but confesses the difficulty of the subject, and does not enter on its discussion. He states that, while at Nassau in April (the fever broke out in July at Honduras) the ship's holds had been emptied, cleaned thoroughly, white-washed, and completely dried and ventilated, prohibiting the notion of want of interior clearness having any thing to do with the production of the disease. Such processes of clearing, perfectly cleaning, and then re-stowing ships of war in the West Indies, with the view of guarding against invasions of fever, are common; but it is a fact, however startling or difficult of explanation, that they are very generally followed, in no long period, by a serious visitation of the disease. What relation there is between the purifying process in question, and the subsequent eruption of fever, if it be an operative relation, may never be satisfactorily known; that the one frequently, generally, follows the other, is certain."

Fever prevailed, too, in the Grasshopper, the Icarus, and the Magnificent; it occurred, also, to some extent in other ships, but, generally, it had little force; and, on the whole, the year 1830, though the loss in some ships was rather severe, cannot be considered unfortunate as regards West Indian fever.

Although the pure inflammation with fever (order Phlegmasiæ) amounted to 1,468 cases, only four deaths, viz. two from inflammation of the lungs, one from inflammation of the bowels, and one from inflammation of the throat, resulted from the whole on board; but 48 were sent to hospital, and 54, as already stated, were invalided; so that the temporary or permanent loss to the squadron was not inconsiderable.

Next in order of frequency were rheumatic cases; after which, inflammation of the lungs, of the liver, and of the throat, were most numerous, many cases of each occurring. Including hæmorrhage from the lungs, there were 36 phthisical cases; of which, 13 were sent to foreign hospitals, 11 were invalided, and two terminated fatally at sea, leaving 10 under treatment at the end of the year on board.

The catarrhal cases amounted to 556, of which four were sent to hospital, and one proved fatal at sea.

There were 47 cases of dysentery, four of which were sent to hospital. No one ended in death, and none required change of climate.

Diarrhœa was, as might be expected, frequent. There were many cases of common cholera, one of which was fatal, the only death which happened in the order Spasmi, though 488 cases were treated.

There were 139 cases of all forms of venereal disease, viz. 64 of syphilis, 39 of gonorrhœa, 7 of stricture in the urethra, 14 of bubo, and 15 of diseased testicle. Of the two last forms of disease all might not have a venereal origin, but there is little doubt that most of them had. Nine of the syphilitic cases were sent to hospital; one of the gonorrhœal, and a case of bubo were invalided, all the other men affected being cured on board: the detriment sustained by the disease was therefore not great: most of it was contracted at Halifax, and other northern parts. In the West Indies syphilis is comparatively rare, nor is gonorrhœa very frequent.

The ulcerative cases amounted to 309—rather a large number; many of which, proved tedious and difficult of permanent cure; 20 of them were sent to foreign hospitals for treatment, and six were sent home invalided. The disposition in some vessels to the ulcerative process, was great, almost every slight accident, abrasion, or small abscess, assuming that character.

*Year* 1831.—The squadron enjoyed a high degree of health, or, at least, a low degree of mortality. In few parts of the world, says the Reporter, comprising equal numbers and equal spaces, will there be found less annual loss of life than happened on that station in the year 1831. Out of a mean force of nearly 3,000 men, only 14 died at sea, and 20 in hospital, a low number in itself—low when compared with other naval stations enjoying higher reputation as to climatorial influence on health, and very low when compared with its own results on life at some other times. When exempt from the peculiar, destructive fever, the product of their soil and climate—and, fortunately, they have frequent and sometimes long-continued exemptions from general epidemics—the West Indies may be pronounced healthy; at least, they are not fertile in other forms of disease which suddenly destroy life. In the year 1831 only one ship suffered from the fever in question, in an epidemic form, and that not in a severe degree.

The only particular we need allude to is the following:—The Sparrowhawk suffered considerably from continued fever, having had within a few weeks 109 cases, of which six terminated in death on board, and one in Port Royal Hospital, out of 17 sent there. The disease broke out on a passage from Jamaica to Chagres, after touching at the Bocca Chico, the mouth of the strait leading to the city of Carthagena. Fever of the worst form often occurs under similar circumstances, viz., ships, after having lain some time in Port Royal Harbour, proceeding to Chagres, remaining on that coast awhile, and then returning to Port Royal. In what way the climatorial influence of Jamaica co-operates with a similar or separate influence on the Spanish Main, in the production of West Indian fever, may not be ascertained; but a great many instances might be cited to show that there is such co-operation. The fever in the Sparrowhawk was, from the account of the symptoms, the pure endemic product of the West Indies, though not possessing a high degree of force. It may be stated, that there does not appear to have been any relation between the touching at Bocca Chico and the eruption of the disease.

*Year* 1832.—Low as was the rate of mortality within the limits of the West Indian and North American command in 1831, it is still lower in 1832. The more common forms of diseased action were, for the most part, mild and tractable, though some of them occurred in large numbers; and pure West Indian fever, the peculiar product of West Indian soil and climate, separate, and essentially different from remittent and other modifications of endemic fever, scarcely existed. It certainly did not exist generally or frequently, though a small number of isolated cases throughout the squadron, probably occurred. Few, if any, years pass without the appearance of occasional cases, nor is it probable that this year was free from them. When it is absent, or occurs in very small numbers, the West Indian portion of the command is not unfavourable to health; the northern division, though it frequently abounds in catarrhal, rheumatic, and slight bowel complaints, is not productive of violent and destructive forms of disease; and hence, in this, and similar years of total, or nearly total, exemption from West Indian fever, the united squadron exhibits, and will be found to exhibit, favourable returns, as to healthy condition.

Only 25 instances of death happened on board ships, giving the very low ratio of 6·9 per 1,000, though a trifling increase on that of last year. But the proportion of mortality in hospital was smaller this year than last, only 15 men having died out of 277 sent there, making a total of 41 instances of death—the ratio of the entire mortality on the station being no more than 11·4 per 1,000 of mean strength.

The Sparrowhawk, it will be recollected, suffered severely, in 1831, from "West Indian fever." This year she suffered too, but the fever presented some difference of features. The cases amounted to 20, of which four terminated fatally. The ship was anchored in Montego Bay, from the 4th to the 21st of January. A portion of the ship's company was landed for the purposes of protection, the slave population being in a state of insurrection, on a property called Unity Hill. The men occupied sheds on an eminence, at the point where the great river empties itself into the Bay, the land at the base, and some distance inland being a complete swamp. The temperature in the night was as low as 64°, rising during the day to 80°, the people suffering from fatigue, and perhaps from various kinds of excess. No combination of things could be imagined more likely to occasion certain forms of fever: fever soon made its appearance among the shore party, and prevailed to the extent stated above.

Delirium tremens rather obtained this year. All over the West Indies, as well as at Halifax, rum, and other intoxicating liquors, are cheap, and still too generally the object of keen desire among British seamen. When on leave, drinking to excess is yet a common practice, and in spite of the utmost vigilance, spirits are frequently, and by the most singular means, smuggled on board while ships are in harbour. Great pains are taken to prevent this, and there is no reason to believe that there was any relaxation of the preventive measures in 1832; yet there were 10 cases of delirium tremens, two of which terminated fatally at sea. The Reporter observes: the regulation which, some years ago, reduced the allowance of spirits to half the former quantity, was excellent, and has done much good. Whether the allowance might be further reduced, and yet be retained as a regular ration, may be questioned; but there can be no question as to whether commanding officers should have the power of unconditionally stopping the spirits of individuals, on the production of proper evidence, and that more as a remedial, than a penal measure. The practice, too, of allowing every lad as soon as he is rated the full allowance of grog, should be seriously considered. We decidedly agree with the Reporter on the impropriety of allowing lads spirits. They would be far better, in every way, without them.

*Year* 1833.—The health returns are less favourable in 1833 than they were in the two immediately precedent years; yet the difference is not great, the loss sustained is not severe, and the ratio of mortality—little more than one-and-a-half per cent.—cannot be considered formidable throughout a command which comprehends the West Indian Islands and adjacent coasts. The small comparative difference which appears against this year depends upon the operation of malignant cholera. Though there was a good deal of fever, there was little of the "West Indian"—hence the inconsiderable resulting mortality; for there are strong grounds for concluding, that whenever that form of fever prevails, there will be, without underrating the value of right treatment, great loss of life."

The mean force was 3,386. The number of sick and hurt was 5,335, yielding the large rate of 1575·6 per 1,000 of mean strength. Out of the whole, 382 were sent to foreign hospitals, 114 were invalided from various ships, and 25 terminated fatally at sea. Besides 114 cases of invaliding from ships, 21 occurred at hospitals, making a total of 135, and giving the ratio of 39·9 per 1,000 of mean strength. There were 30 cases of death in hospital, which, added to 25 on board ship, make a total of 55 cases of death, and give the ratio of 16·3 per 1,000 mortality of mean strength of squadron. The ratio of mortality, though higher than in the two preceding years, is not high in itself, and is low when compared with many equal periods of time in the same places. The number invalided is greater than in the three preceding years, and is certainly considerable in itself. To lose four men out of every 100 through this channel is to suffer much detriment.

*Year* 1834.—The Reports are not quite so favourable as were those of the three immediately preceding years. The Tables of 1833 gave a higher rate of sickness and mortality than those of 1831 and 1832, and those of the present year are higher than any of the above-named. It is higher also than 1830 in all the tables indicating disease and accidental injuries, and their results, excepting that of mortality. Though the proportion of sick and hurt is greater in 1834 than it was in 1830, the proportion of deaths is less. In 1830 the mean strength of the squadron was 3,326, the ratio per 1,000 of sick and hurt 1524·3, total number of deaths 75, this year the mean strength is 3,636, ratio per 1,000 of sick and hurt 1658·7, total number of deaths 72, showing, though less strikingly than some similar returns, that the rate of mortality has no necessarily close connexion with the amount of sickness. But though the columns of the present year exhibit higher numbers than those of 1831, 1832 and 1833, they are not fitted, either alone or relatively, to excite wonder or alarm, especially in reference to the amount of mortality, of which the ratio is under 20 per 1,000 of mean strength. The increased mortality of this year, as compared with that of the three previous years, depends principally upon the greater prevalence and power of fevers, and partly on a larger proportion than usual of death from accidental causes, six cases of that kind having occurred, three of them from drowning.

There is a statement in this year's Report in reference to the malignant cholera, which, though not unique, is still not unworthy of notice. It may be added to some other and similar ones on record.

Eighteen cases of cholera appeared in the President; only 3 of the 18 men attacked by it died. It apppeared in the ship in August, having been found to exist in the town (Halifax) a short time before. The ship was anchored close to the town. The ship's company, after the existence of cholera in the town was ascertained, were not allowed to go on shore, though one man, who had been employed at the admiral's house, near which place the disease did not exist, was sent on board the ship. Two other vessels of war, lying near the President had neither of them a single case of cholera. It has been omitted to state whether those vessels were further from the shore than the President, and what position they occupied in relation to the part of the town in which the disease broke out, and then chiefly prevailed. Four days after the appearance of cholera in the President, she was moved from her original moorings, near the dock-yard, to a point three miles distant in the harbour; after the change of place, no case of the disease occurred. The rifle brigade had suffered severely from the same disease some time in barracks, when they were marched to, and encamped at the bottom of the bay, a distance of several miles; with the change of place, the disease in them also entirely ceased. The same favourable results followed the removal of the 96th Regiment from barracks, where the disease was committing great ravages, to an eminence, distant less than a mile.

*Year* 1835.—The mortality in the united West Indian and North American Squadron was much greater during the year 1835, than in any previous year embraced by this Report, that is, from the year 1830 to the present.

"The mortality has been progressively increasing through a period of five years in the united squadron, the rate of increase being proportionate to, and dependent on increase in the force of febrile disease. During the first three years the increase was inconsiderable, and therefore, though it probably had much meaning in an etiological point of view, was not likely to attract much observation; but between the two last years, 1834 and 1835, the difference was striking; the number of deaths, on comparing the latter with the former, being nearly double. Progressive increase of this sort in West Indian fever, and in other endemic diseases, during a number of consecutive years, tending to and terminating in a general distribution of its agency, is often observed. It may be represented, in a general way, as taking the following course. After the lapse of a year or two, in which it can scarcely be traced, it occurs rarely and separately during another year or two. During another similar period it will arise more frequently, but still with considerable intervals of time and space; then, connected perhaps with a particular state of weather, it bursts forth simultaneously at various points of the island and its harbours; the endemic becomes epidemic, attacking at once ships, regiments, and citizens, and carrying off great numbers. After such a course, though it may linger for a little with greatly reduced force, in some places, a period, longer or shorter, of exemption ensues. The cause of the disease appears to be exhausted for the time, the stock accumulated, so to speak, worn out; and a certain period is necessary to its reproduction."

The Reporter asks the meaning of this. But, as neither he nor we can tell, we need not indulge in speculations on it.

The mean numerical force for the year was 3,199. The number of sick, being as usual a large proportion to the number employed, was 4,724; the number of cases sent to foreign hospitals was 505; the number invalided was 129, viz. 166 from ships, and 23 from hospitals; and the number dead was 120, viz. 38 on board various ships, and 82 in hospitals. The first, the number of sick, are at the rate of 1476·5 per 1,000; the second, or hospital cases, are at the rate of 157·8 per 1,000; the third, the number invalided, are at the rate of 40·3 per 1,000; and the last, or number of dead, are at the rate of 37·5 per 1,000 in reference to the mean strength.

The great and striking difference in the Tables of this year, as compared with the Tables of the five former years, however, is in the increased rate of mortality. The increase is occasioned exclusively by the increased frequency and force of fever, that disease being the cause of nearly five sixths of all the fatal issues; while other causes of death are less numerous than usual. The fevers happened altogether in the South or West Indian division of the command: so that, were the numbers employed in the Northern division deducted from the numerical force of the entire squadron, the rate of mortality, though not so formidable as it often was many years ago in the West Indies, would this year be high.

The Reporter mentions some circumstances connected with the West Indian Fever as it appeared in the Vestal, which are not unworthy of attention. She was a small frigate-built ship, with a complement of 180 persons.

"She had 167 cases of primary attack, of which 139 were sent to hospital, and three terminated in death on board; 25 fatal terminations took place in hospital. The disease made its appearance on board early in March, in Port Royal Harbour, without much severity or frequency, and without exciting alarm; but it speedily became more prevalent, and ere long general, with increase of intensity, and fatal force. On the 30th of the month the ship was moved from Port Royal Harbour, where, with two short exceptions, she had lain nearly four months, and anchored a mile and a half off, near a Key—a small, sandy, uninhabited island. The change was effected in the belief that the disease depended on something derived from the harbour, and that it would therefore cease soon after moving the ship to a distance. It however went on, after the change of position, with unimpaired frequency and force. It ought to be stated that the fever was not connected with want of cleanness, of ventilation, or of pure air, in the common sense of the phrase; for the holds had lately been cleared, cleaned thoroughly, and white-washed; windsails were diligently employed, and chloride of lime was sprinkled on the decks abundantly. Another circumstance ought to be stated, not for its rarity, but for its frequency, and its importance in considering the cause of this disease, more especially in ships. It is this; almost every person who joined the Vestal during the prevalence of fever was affected by it, but no person leaving her under the disease communicated it to another, in another place. And so it happens, if not universally, almost universally. Nearly every man who joins a ship in such a condition has the prevalent disease sooner or later; but no number of persons taken from such a ship, labouring under the disease in any stage, or in any force, and placed in a situation where the disease does not exist, though in the centre of a mass of healthy people, can excite it in a single instance. An accumulation of such facts—and there is a large accumulation—decides the question of the contagious power of the fever in the negative absolutely." The vestal sailed for Bermuda on the 28th of April, and the disease finally ceased on the 8th of May, in the 27th degree of north latitude.

Another statement, relating to the Forte frigate, is also worth noticing. There were 123 cases, five of which terminated fatally aboard; eight were sent to Port Royal hospital. The disease, though causing little comparative mortality, and in most instances mild, appears to have been the true West Indian fever. It is noticed chiefly on account of two circumstances, both of which are so constantly found connected with the disease on board ship, one of them antecedent to, and the other concurrent with, its eruption. The antecedent is the cleaning the holds, and otherwise most completely cleaning the interior of ships. The Forte had been five weeks in Port Royal Harbour, undergoing such a process, the people meantime living on board the Magnificent. She sailed for Vera Cruz on the 11th of June, and three days after the disease broke out. The concurrent circumstance is the place in the ship where the disease first appears, viz. the immediate neighbourhood of the pumps. So it was in this ship, so it was in the Rainbow, and so it will be found in a great majority of cases to be.

"The Dee (steam ship) had 47 cases of fever, all of which were sent to hospital, where eight of them proved fatal. There is nothing very remarkable either in the number affected, or the resulting mortality; but there is something remarkable in the progress of the disease, as to time, in the ship. Instead of occupying, as it commonly does, a month, or two or three, the disease, though not equally, embraces the greater part of the year. Something similar is observable in the steam ship Rhadamanthus. More time and a larger field of observation are required to settle the point; but if such diffusion, as to time, in the operation of the cause of fever, were found to hold generally in steamers, it would be interesting to inquire whether it was connected with the steam, or, rather, with the heat which produces it."

What occurred in the Rainbow, may be alluded to. The ship had lain in Port Royal Harbour six weeks previous to the appearance of the fever aboard, which happened in the middle of July; it was not extinguished till the close of October. In the surgeon's journal it is stated that the ship's company were removed from the Rainbow, after the fever had been some time in progress, into the Magnificent "to have the holds cleaned, fumigated, ventilated, &c."—"the removal to the Magnificent seemed to check it, but after a short time it commenced again, particularly with those people who were obliged to keep watch on board (the Rainbow), the marines, midshipmen, &c. though not sleeping there, but merely going to keep their watch, and returning when it was over." The Reporter observes that—"the removal of the people from a 'sick ship,' in a case like the present, is a point of great interest both practically, and doctrinally. If the removal were to a place in itself free from the cause of the disease, the writer of these remarks believes that the remedy would be complete, and that with it the disease would entirely cease, excepting in such persons as had imbibed its cause before they left the 'sick ship.' In the case of the Rainbow, the marines, &c. who returned to her for the discharge of certain duties, and who are said to have suffered particularly after being moved to the Magnificent, were evidently exposed by such a return, to a certain extent, to the original cause of the fever."

*Year* 1836.—"The mortality," says the Reporter, "in the united West Indian and North American squadron was remarkably small during the year 1836, in itself, and as compared with that of 1835. It will seldom be found so inconsiderable on that station, and on others occupied by the royal navy; it is often greater in the civil population of Great Britain, among persons of corresponding ages, and in the absence of severe and spreading epidemics. In 1835 the mortality was nearly four per cent.; in 1836 it was under one per cent. The difference as to fatal results, depended on difference in the prevalence and force of fever, being great in the former, and trifling in the latter. The cause of West Indian fever, the peculiar product of the locality, which, through some preceding years, had been becoming progressively more generally diffused, and more forcible—from whatever source, or through whatever agency—had in the year 1835 acquired its highest degree of force and development; the process by which it is formed was completed; it was evolved and exhausted; and hence the extreme rarity, or absence of the disease in 1836. In such a case the inquirer is compelled to judge by the results. It would have been impossible to have foretold, during the progess of the disease in 1835, that it would not have extended to, and become more general, and more violent, in 1836. There were no means of ascertaining that the cause had then reached its maximum—the period of maturation—and that it would consequently cease to act, or would act seldom during the following season. In this, as in other endemic epidemics, the cause is formed, evolved, and becomes operative in different degrees of force and frequency at different periods of recurrence. It was more generally diffused, and more destructive at Jamaica, in 1825, than in 1835, as it had also been at some former periods."

The Reporter states that the Carron and Alban, two steamers, suffered more from fever, in proportion to their complements of men, than any other ship on the station. The evidence, so far as it goes, is in favour of the opinion that the fever in those vessels was the West Indian. The Reporter indulges in a curious vein of remark. He says:—

The vessels being steamers, and the season not generally productive of the disease—did the heat requisite for the production of steam, precipitate the evolution of the cause. No degree of heat alone can excite West Indian fever, or any other fever in the right sense of the word. But the question is—did the heat here, co-operating with the endemic morbific influence of the spot, so act upon the structural parts of the vessels, as to call into action the cause of the disease, which, but for that high heat, would not have been evolved then, and, under other circumstances, might not have been evolved at all? The question is important in itself, and involves another still more important, to the following effect. If, as there is reason to believe, a high degree of heat is necessary to the production of the disease in ships, in so far as it is derived from an *internal* source; and if high degrees of heat have the power, as there is also reason to believe, of precipitating, and more speedily perfecting the cause of the disease, as acting more energetically on their structural parts, contributing the results of certain decomposable materials to that effect, might not the knowledge of such facts be acted on for the prevention of the disease, in so far as it has an internal origin, and is occasioned by changes effected in the materials of the ships themselves? The preventive measure which suggests itself is, the application of a certain degree of heat for a certain time to the wooden materials of ships, or perhaps more appropriately to the wood of which they are to be constructed—say a temperature of 160 degrees for a month. It is reasonable to infer, that such a degree of heat, applied for such a time, would dissipate the decomposable matters, on which West Indian fever on board ship, in many cases, must in some way depend. It does not appear probable that the process would act detrimentally on the woody fibre, and impair its durability; nay, it might improve the one, and increase the other. That, in many cases, and the worst cases, West Indian fever is essentially connected with some agency in the interior of ships, altogether independent of personal communication, or collections of extraneous matters, all discriminative experience shows.

As in the Report from the South American station, we are presented with several Tables for the seven years, from 1830 to 1836. The *first* shews the total number of cases; the number of all diseases and injuries, in classes; the number of cases sent to hospital, invalided and dead; with the ratio of each per 1,000 of mean strength.

The Reporter observes, in reference to this Table, that the annual rate of mortality, on an average of seven years, was, from all causes, diseases and external injuries, 19·6 per 1,000 of entire force; and from disease, independent of injury, 18·1 per 1,000. It includes, besides the mortality on the station, the deaths which occurred in home hospitals, from disease contracted within the limits of that station; so that 33 invalids having died in English hospitals, the mortality from disease on the station was in the ratio of 17·5 per 1,000 per annum.

Comparing the mortality in and from this command with that resulting from service in South America, it will be found to be, from all causes of death, more than double; from disease, distinguished from external causes, as 18·1 to 7·7, the proportion of death from disease, in relation to accident, being much higher in this command, than in the other.

Yet something more than doubling the mortality of South America is not productive of formidable results; nor in this command, the greater part of which is constituted by the West Indies, and which had epidemic cholera, though to trifling extent, added to the ordinary causes of death, will mortality at the rate of 18 per 1,000 per annum appear excessive. When the nature of West Indian fever is considered, and the rate at which it sometimes prevails, and proves fatal, the rate of mortality will rather, and in opposition to generally received impressions, appear small. The most fatal year was 1835, when the ratio of deaths, from all causes, rose to 37·5 per 1,000 of force, and from fever to 30·6. The following year it fell to 9·2 per 1,000 of force, from all causes, including external injuries.

The annual ratio per 1,000 of sick and hurt was 1486·3, the total number placed on the surgeons' lists being 34,982, and the numerical force of seven years 23,531.

The number invalided was also high, viz. 926, being in the ratio of 40 nearly per 1,000 per annum of force.

The reduction of active force in the command was, from invaliding and death, nearly 59 per 1,000 per annum; so that, though the loss by death, keeping in view the nature of the climate, was small, the total reduction of number was considerable.

Comparing, he adds, these ratios of attacked and invalided with those in South America, it will be seen that they are considerably higher, though that of attacked, considering the great difference in mortality, is less so than might have been anticipated. The annual ratio of sick and hurt per 1,000 in the South American command was 1310·7; that of invalided 28. Hence, while the reduction of force, by death and invaliding, in the West Indies and North America, was in the ratio of 58·9 per 1,000 per annum, in South America it was only 36·9. Had South America possessed the advantage of hospitals, it may be inferred that the loss, by invaliding, would have been considerably less than it actually was.

The *second table* shews the total number of cases, the number of all diseases and injuries, in classes; the number invalided and dead; with the ratio of each per 1,000 of attacked.

The most striking result brought out by this Table is the mortality from fever, especially when compared with the mortality from the same cause in South America. In this it is in the ratio of 11·2 per 1,000 per annum of strength; in that it was 1·3 per 1,000 per annum. The mortality from fever was therefore nearly nine times as much in this command as in the other. Whereas in South America it was as 1 to 7; in this command it is considerably more than half of the total mortality, which amounted to 19·6 per 1,000 per annum, though malignant cholera, a new disease here, occurred to augment the mortality from other causes. Had the command been confined to what is commonly called the West Indies, to the Carribean Islands, and adjacent shores of the Spanish Main, and Gulf of Mexico, and to the Bahamas, not united, as it was, with North America, the rate of mortality would have been much higher; it may be assumed that it would have risen to 15 per 1,000, by fever, of the employed annually.

The mortality from fever was confined to the southern, or West Indian, portion of the command; on the other hand, a large proportion of the mortality from original inflammatory disease was in the northern, extra-tropical part of it.

The total number of cases treated under the denomination of fever was 4,932; but the great majority were trivial febrile attacks. The aggregate force of the seven years being 23,531, the annual rate of febrile attacks was 209·6 per 1,000. Of the whole number affected, 1,124 were sent to hospital for treatment, 70 on account of imperfect recovery were invalided, and 264 died. The annual ratio per 1,000 of the first is 47·8, of the second 2·9, and of the last, as already stated, 11·2. Of the fatal cases, three were in home hospitals; the subjects were invalided for the effects of fever, but the name of the primary disease was retained; hence the anomaly of deaths in England by West Indian fever.

There were 519 cases of inflammation of the lungs, and their membranes; of which, 99 were sent to hospital, 26 terminated in a state which led to invaliding, and 22 ended fatally. The annual ratio of attacks was 22 per 1,000, of hospital cases 4·2, of invalided 1·1, of dead nine, of mean force. Comparing these ratios with those in South America, it appears that, while that of attacked is lower, that of invalided nearly the same, that of dead is nearly double. It is probable that frequent sudden transitions from high to low degrees of temperature, in this command, contributed to increase of severity, and fatality in result.

The mortality from inflammation of the liver was less by a half in this station than the South American. The annual ratio of deaths from disease of the liver was one in 5000 of the employed.

Under the head of "consumptive diseases of the lungs," 114 cases were treated; of which, 52 were sent to hospital, 56 were invalided, and 44 terminated fatally, 28 on the station, and 16 in home hospitals. The ratio both of attacks and of deaths is much higher than in South America; in this command the attacks are 4·8, the deaths 1·9, in that the attacks were 3·2, the deaths 1·5, per 1,000, per annum, of strength.

After fever, consumption was much the most fatal form of disease in the command; after that again, inflammation of the lungs was most fatal.

Two hundred and eighty-seven cases of dysentery were treated; of which 42 were sent to hospital, 15 were invalided, and six ended in death. The ratio of attacks and of deaths is low, the last strikingly so, when it is considered that a large portion of the command is within the tropics. The mortality is not a third of what was suffered from the disease in South America, though it was not severe there.

The ratio of attacks of *delirium tremens*, as well as of deaths, is nearly double of what occurred in South America. It is remarkable that a great portion of them were in one year, 1832.

A Table is given of the *frequent, but not often fatal diseases.*

Numerous, says the Reporter, as were superficial inflammations of the extremities in South America during the seven years of this Report, they were greatly more numerous in the West Indies and North America. Whereas they were in the ratio of 166·9 per 1,000 per annum of force in that command, they were in the ratio of 228·3 in this, the number treated being 5,372, and amounting to nearly a sixth part of all the cases placed on the surgeons' lists. Out of the whole number, one, from change of character, or superinduced disease, terminated fatally.

Though the rheumatic cases were less numerous in this command than in the other, they were much more detrimental to force. Here the ratio of attacked was 69 per 1,000 of force annually, there it was 72·3; but here, in addition to 133 sent to hospital for treatment, 121 were invalided; whereas there no more than 24 were sent to hospital on the return of ships to England, and 43 invalided. In South America the annual ratio of invalided was 2·5 per 1,000 of force; in the West Indies and North America it was 5·1, or more than double. It is difficult to account for such difference in results from this disease; the difference of structure in the two commands, as to temperature, suggests itself; but then it does not appear why it should not act in augmenting the frequency, as well as the severity, of attacks in the West Indies and North America. The same number of cases terminated fatally in both commands, viz. four; the ratio of death to force is, of course, a little lower in this command than in South America, while in relation to attack it is higher.

The ratio of attacked by catarrhal disease was much higher in this command than in the South American; it was 181·8 per 1,000 per annum of force here, while there it was 139·8. In the South American there were 25 cases of invaliding, in this there were only nine; but in that command there was only one fatal case, while in this there were three fatal cases.

The ratio of attacked by diarrhœa was also much higher in this command than in the South American; here it was 110 per 1,000 annually of force, there it was 80·6. Dysentery, on the other hand, was much more severe in the South American command than in this.

An Appendix of Tables concludes this, as it did the former Report, and is sufficient evidence of the indefatigable industry of the compiler.

We must defer to our next number the conclusion of this Report. In the mean time we would direct our readers' attention to it, and recommend them to add the facts that it embraces to those contained in the Army Reports. Altogether, those facts are extremely valuable.

